# Assessment of High Thermal Effects on Carbon Nanotube (Cnt)- Reinforced Concrete

**DOI:** 10.1038/s41598-018-29663-5

**Published:** 2018-07-26

**Authors:** Hala Elkady, Ahmed Hassan

**Affiliations:** 1 Civil Engineering Department, National Research Centre, Giza, Egypt; 2 Civil Engineering Department, Beni-suef University, Beni Suef, Egypt

## Abstract

This paper presents the outcome of a project funded by Beni-suef University targeting the assessment of the addition of carbon nanotubes (CNTs) to reinforced concrete beams on exposure to elevated temperatures. Tests were carried out in compliance to ASTM E119-95a. Besides, pre and post their exposure to elevated temperature tests, the maximum bending capacity of the beams are evaluated. Standard reinforced concrete beams are cast- with and without Carbon Nanotube (CNT). Tests were performed at two temperature levels 400 °C and 600 °C working around temperature ranges expected to have significant effect on concrete endurance, using both one and two hours exposure. Results proved a positive effect for adding CNT to beams at room temperature. This improvement is slightly affected at 400 °C exposure for 2 hr. On the other hand, exposing CNT beams for 600 °C for two hours reduced the beams capacity by 14% compared to a similar reinforced beam without CNT. It is worth notice that CNT was not burnt but suffered de-bonding. Finally, this investigation implies that CNT can be used as an enhancing element to concrete ductility, with no deterioration in other mechanical characteristics on exposure to drastic thermal conditions.

## Introduction

Concrete components are not perfectly bonded, consequently, pre-cracks and pores often exist in hardened concrete^1,2^. Many reinforcing techniques are based on fillers to fill in the pores, connect the components and transfer stresses in concrete. Fiber-reinforced concrete (FRC) is a product out of this bridging concept. Those fibers length scale ranges from micrometers to centimeters, but do not reach Nano voids filling. Nano-additions can modify the packing of Ordinary Portland Cement Concrete, improve its hardened performance^3^. Several studies on concrete with Nano silica (NS) and carbon Nano fibers (CNF) have been carried out in order to identify their advantages in the fresh and hardened performance^4,5^. Theoretically, Nano-fibers should be good candidates to control early age cracking, as the addition of CNF increased 30% flexural strength of fluid pastes and it does not control cracking and even increased it^6^. Carbon nanotubes (CNTs), known for superb material properties and the elegant structure^7–9^, are of great potential to be extensively dispersed into cement filling up the pores and modifying the structures at the Nano scale. In order to mitigate early age cracking, small amounts of micro-fibers can be incorporated^10,11^. In order to gain superior comprehension of interaction between CNTs, C-S-H and resulted a systematic evaluation, mechanical enhancement on the method of the CNTs in the concrete system and CNTs bring about the mechanical enhancement necessary a series of reviews can be found^12–18^.

It is expected that using carbon nanotubes shall enhance the concrete mix behavior. It is well known that Concrete as a cementitious composite is held together by a complex network of nanoparticles known as calcium silicate hydrate (CSH). Due to their Nano-scale characteristics, CNTs shall interact most intimately with CSH. The large surface energy, and the large number of atoms present at the nanotube surface shall promote the formation of an interface with C-S-H. Thus, leading to optimum condition for stress transfer. Carbon nanotubes have high flexibility, and are capable of bending in circles, thus, forming bridges crossing the micro- and Nano-cracks developed in the cement composites. CNTs interact with the C–S–H, generating bridges that enable the CNTs to increase the strength of the cement composites, and stitch the concrete crack^19^. Such interfaces will act as crack blunting mechanisms at the very nascent stage crack growth. CNTs can be widely distributed in the concrete paste; in addition, interaction of CNTs with the paste will be more intense than that of the larger fibers.

Multi-scale modeling techniques are developed and applied to simulate material behaviors of CNT-reinforced concrete^20^. Employed methods mainly involve continuum approaches such as mesh less method^21^ and finite element method^22^. Discussions about fire safety issue are rare. Structural void is a critical concern about strength of solid materials such as epoxy, concrete and bonded composites^23–25^. Adding fibers to bridge voids or cracks is an effective way to mechanical enhancement^26–29^. The materials interface can be quantified by its strength defined as bond quality or adhesion strength^30,31^. The bond quality can be measured by multi-scale experiments^32,33^ and predicted by molecular dynamics simulations^34,35^. It has been revealed that the bilayer materials interface is typically the weakest part in a composite system and is susceptible to external conditioning. In steel bar reinforcement, corruption that degrades bond quality is also a necessary concern^36,37^. Nanostructures of the fiber-cement interface^38^ have been found to be important for adhesion strength. When concrete remains exposed to fire for a long period, losses of its mechanical properties occur^39^. In the case of unprotected concrete, the mechanical properties decrease dramatically when the concrete is exposed to a high temperature^39^. A reduction in the fire resistance rating was observed in the reduced cross-section via the good protection layer during elevated temperature exposure tests^39,40^. On the other hand agglomeration has to be avoided during mixing^41,42^. This article will focus on the effect of elevated temperatures on the CNT reinforced concrete beams. Mechanical characterization is performed pre and post exposure to 400 °C and 600 °C for one and two hours.

## Experimental program

### Test specimens geometric and materials configuration, and investigated parameters

**Figure 1:**
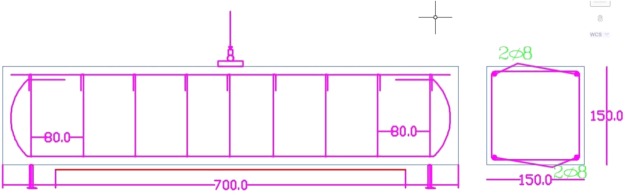
Geometry and steel reinforcement for tested beam.

The specimens used were 10 reinforced concrete beams with rectangular cross-sections with a size 150 mm (width) × 150 mm (height) × 700 mm length. Typical reinforcement pattern is used in all beams: 2 bars 8 mm diameter for both top and bottom reinforcement. Stirrups of 8-mm-diameter are used for shear resistance, and are spaced at 8 cm centre to centre as shown in Fig. 1. Nanotubes with an optimized percentage of cement weight^42^.

**Figure 2:**
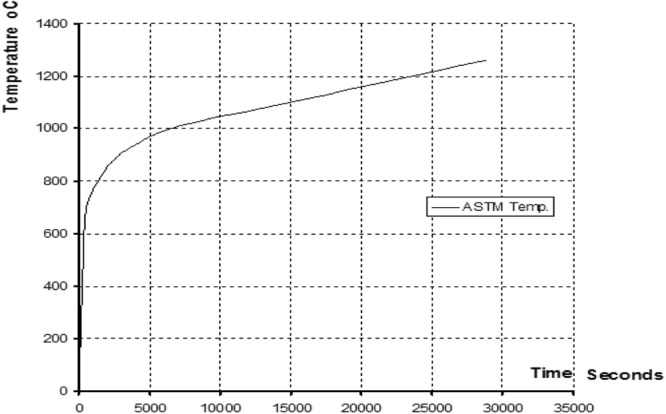
ASTM 119 fire exposure testing curve^43^.

Special attention is drawn to mixing procedure, to avoid de-agglomeration problems, Extensive earlier work^40,41^ investigated the optimization of mixing procedure, and thus, sonication was adopted to ensure proper dispersion. The experiment will be divided into two parts. The effects of CNTs on the flexural and shear strength of normal strength concrete were studied. In this experiment, five groups of specimens will be tested under one point loading to determine the flexural and shear strength of concrete. Each group will consist of three rectangular specimens of dimensions 150 × 150 × 700 mm. The first group will act as the control group, with no CNTs, and the other groups will contain 0.05% of CNTs from cement weight. The following table shows the details of the experiment. Optimized samples from above experimental programs will be thermally evaluated according to ASTM 119^43^ fire exposure testing curve Fig. 2. Specimens will be tested under simulated fire temperature. All tested specimens will be exposed to post fire mechanical tests, to evaluate the degree of loss of strength due to fire exposure. The tested specimens were exposed to different temperature 400 °C and 600 °C for one hour and two hours. Hence, beams were left to cool gradually for two days. After fire exposure, the specimens undergone three points flexural test, to evaluate the reduction in their flexural capacity resulting from high temperature exposure. Codes of the tested beams with different testing parameters are shown in Table 1.

**Table 1:** Specimens coding and elevated temperatures tests.

Specimen Code	CNT	Temperature	Fire test duration
Room	0	25	N/A
Room CNT	0.05%	25	N/A
400 1 hr	0	400	1 hr
600 1 hr	0	600	1 hr
400 2 hr	0	400	2 hr
600 2 hr	0	600	2 hr
400 1 hr	0.05%	400	1 hr
600 1 hr	0.05%	600	1 hr
400 2 hr	0.05%	400	2 hr
600 2 hr	0.05%	600	2 hr

### Material properties and concrete mix preparations

**Figure 3:**
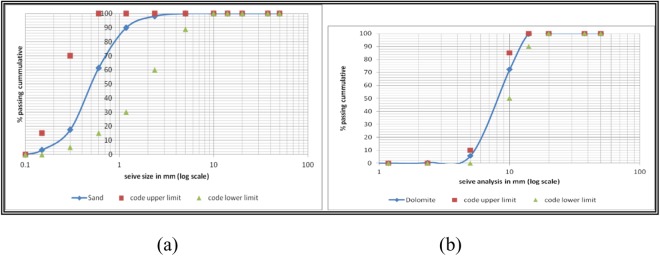
(**a**) Fine and (**b**) Course aggregates Sieve analysis.

**Figure 4:**
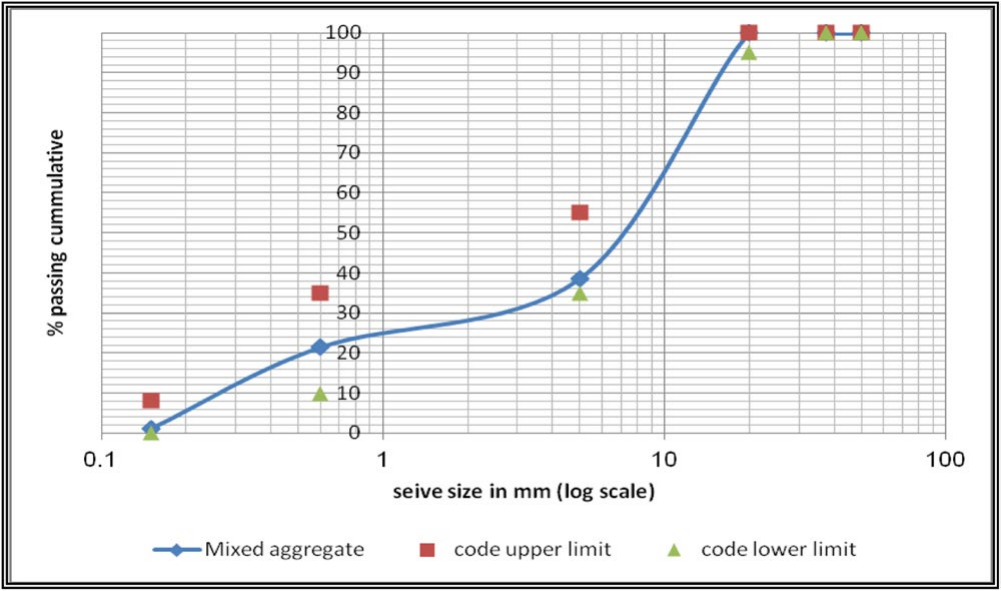
Design mix -Mixed aggregates Sieve analysis.

Ordinary Portland cement (OPC) - ASTM Type I- is used in all mixes, along with natural aggregates and tape water. Concrete design compressive strength is 30 MPa. Mixing was performed using a concrete rotating drum mixer of 0.125 m^3^ full capacity. Sand, dolomite, and cement were dry mixed first. Figures 3, 4 show the sieve analysis of fine, and coarse aggregates separately, as well as the mixed aggregates analysis as compared to standard limitations. Sika Fiber is a monofilament polypropylene fibre for use in concrete mixes, used to reduce the tendency for plastic and drying shrinkage cracking. Besides, it improves abrasion resistance, reduces water migration, improves durability, reduces spalling, and increases the impact resistance of concrete even at early age. Next, water was gradually added while mixing to ensure the homogeneous of the mix. For each mix, three samples of 150 mm cubes with dimensions, and three standard cylinders (150*300 mm) are cast. ACI specifications were followed in the casting and curing system. The mix proportions are reported in Table 2.

**Table 2:** Concrete mix proportions.

Fc (MPa)	w/c ratio	Cement (kg/m3)	Coarse Aggregate (kg/m3)	Fine Aggregate (kg/m3)	Sika Fiber(G) (kg/m3)
30	164	335	1195	644	0.9

**Figure 5:**
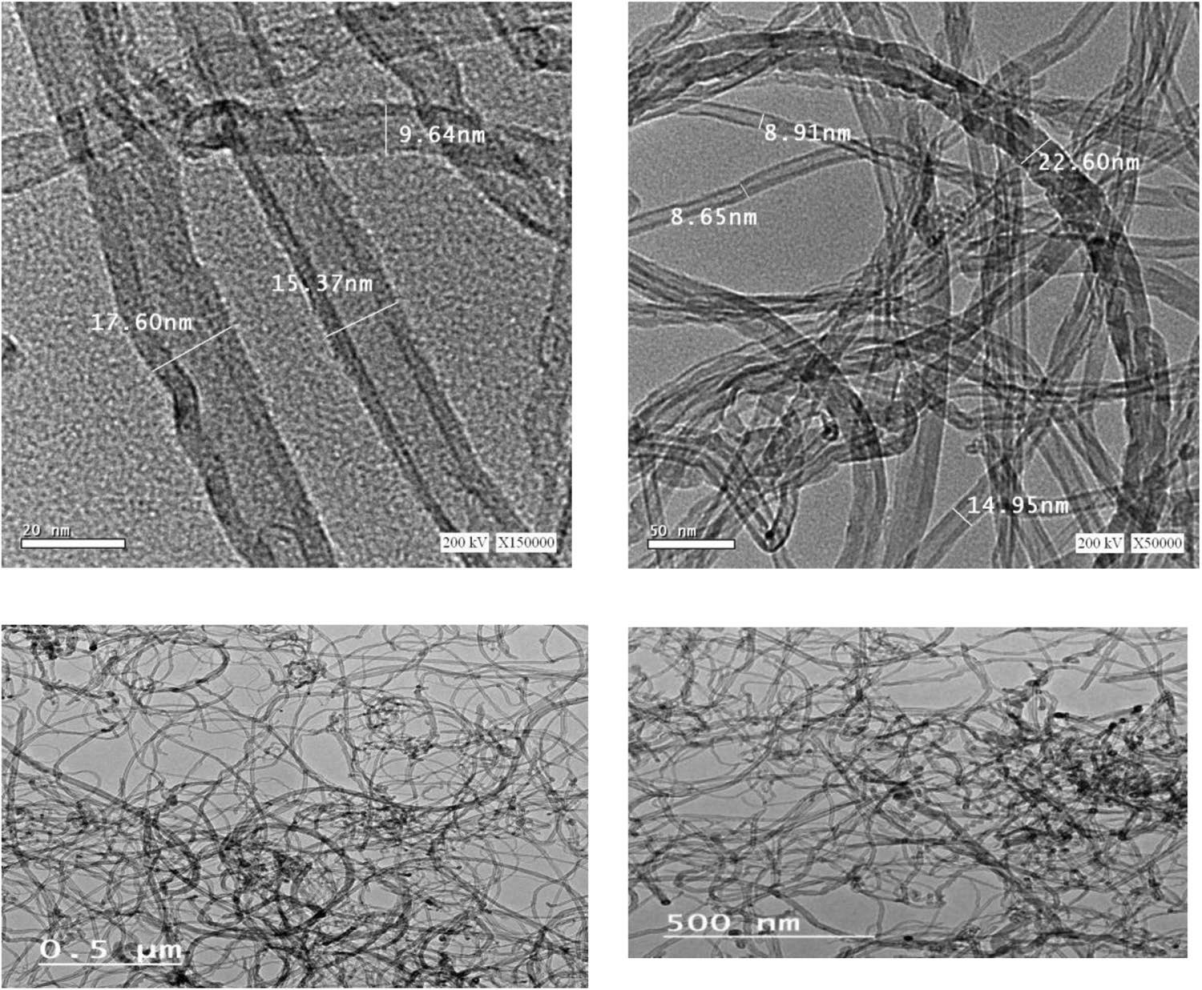
Transmission Electron Microscope Micrograph.

Constant quantity of CNT fibres - 0.05% from cement weight- was added to the design mixes. Multi-walled carbon nanotubes (MWCNTs) are hollow tubular channels prepared by an Egyptian team in Beni Suef University, all technical data is detailed in previous publication^44^. Figure 5 shows the HRTEM images of the prepared CNTS, uniformity in prepared multi-walls is clear, the images with an average diameter was in the range of 8–22 nm.

**Figure 6:**
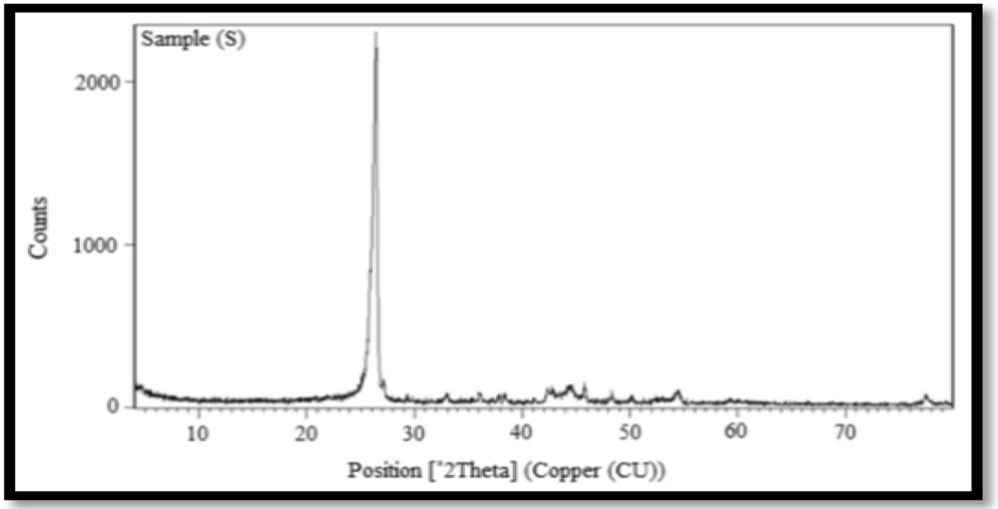
XRD of CNTs.

**Figure 7:**
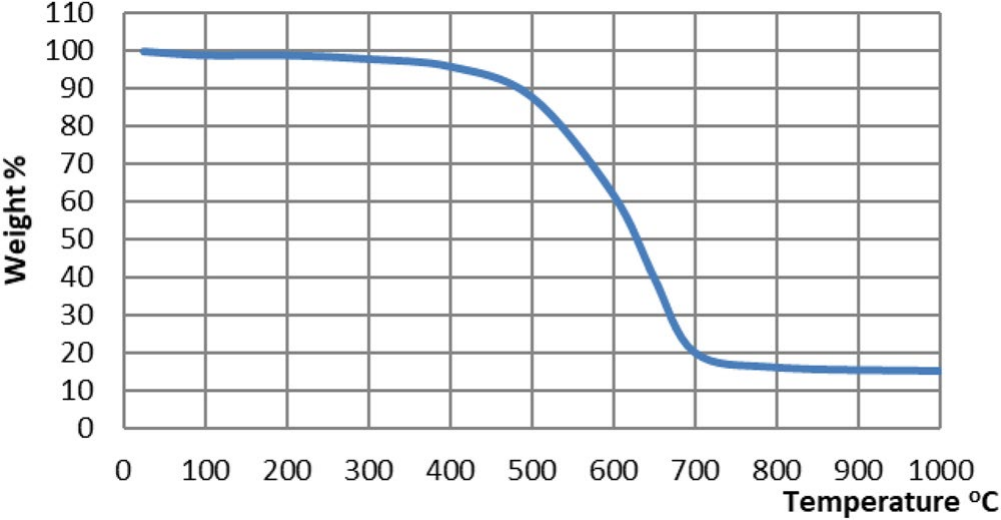
TGA of CNTs.

Figure 6 shows the XRD pattern of the synthesized CNTs, in which the strong and sharp reflection peak was found at 25.76°, this reflection revealed the graphene sheets were nested together to form multi-walled nanotubes. The thermal stability of the prepared MWCNTs was analysed by TGA Fig. 7 in a temperature range of room temperature to 1000 °C. Results revealed that the nanotubes started to oxidize at a high temperature of approximately 500 °C.

### Experimental Program Setup and Testing

**Figure 8:**
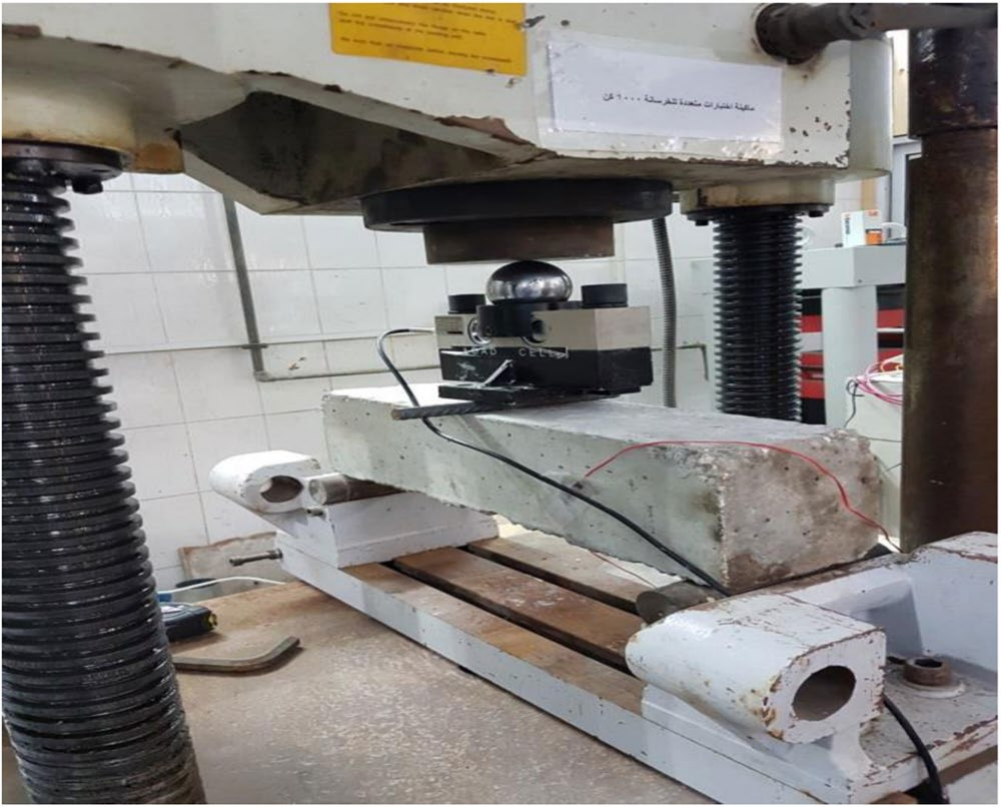
Three points loading flexure test.

Three points flexural tests are performed using a 1000 KN universal testing machine, while elevated temperature tests are conducted using a 1200 °C electrical furnace. Vertical displacements of the tested beams are recorded using a linear variable differential transformer at the middle of the beam, whereas dial gauges were used to measure the vertical displacement at the same location. Strains are monitored at two different locations expected to undergo critical flexural strain and the other at the maximum shear as shown in Fig. 8.

## Results and Discussion

Flexure tests results are displayed and compared for both CNT and RC beams. Pre and post-thermal exposure samples are tested to evaluated the loss in flexural capacity of both concrete beams types due to these high ranges of temperature exposure.

### Three points flexural load tests on CNT and RC beams – pre and post thermal tests exposure Crack Patterns

**Figure 9:**
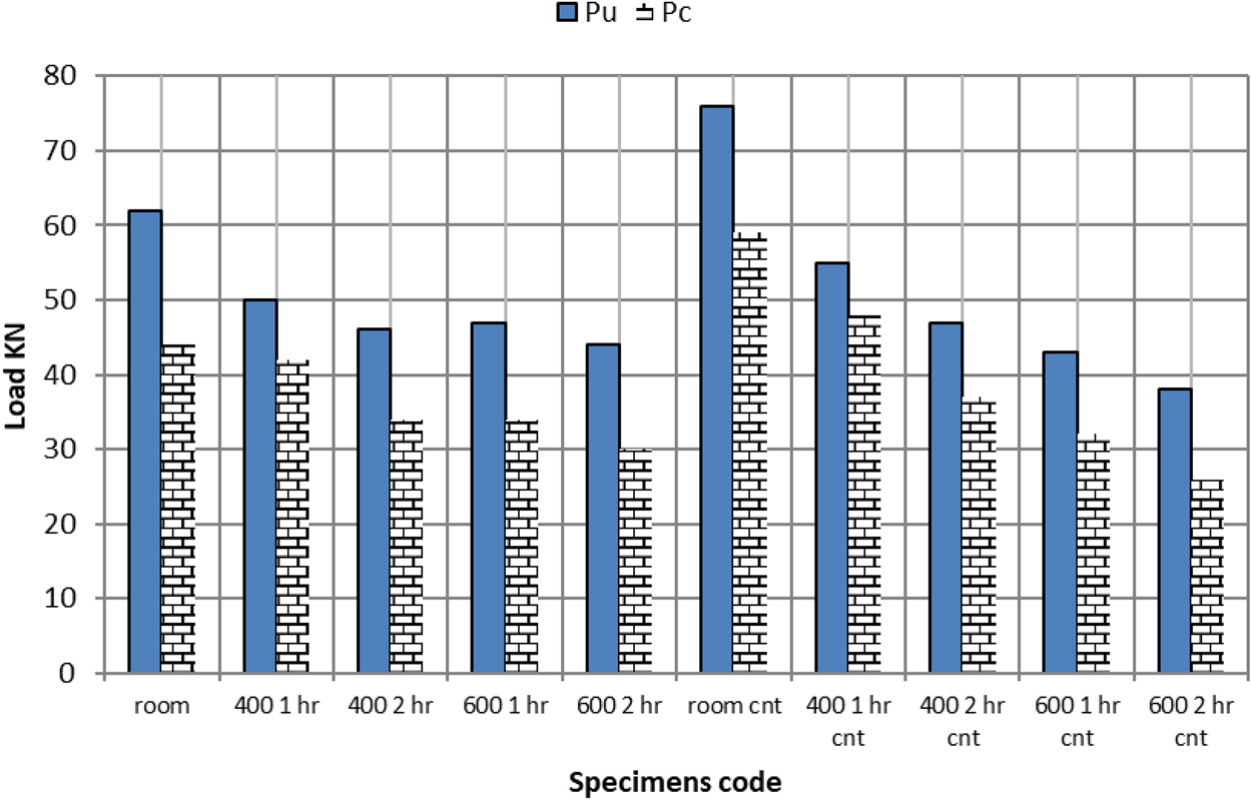
Ultimate and cracking loads for beams under different temperature and durations.

First crack versus loads are displayed in Fig. 9. CNT -RC beams exhibited a higher crack resistance compared to regular RC- beams, as the first crack started at 58 KN compared to a value limited to 43 KN, respectively. This goes in good agreement with previous literature^27^, as CNTs can be spread widely in concrete mix; thus, the interaction of CNTs with concrete will be more intense than using larger size fibres^8^.

Exposure to 400 °C for 1 hour does not have a significant effect on the first crack load, while increasing the fire exposure to 2 hours duration reduced the first crack load to 0.77 of the RC control beam at ambient temperature. Similar reduction is noticed for 600 °C exposure for 1 hour. On the other hand, increasing thermal exposure duration of the 600 °C for 2 hours lead to an earlier observation of the first crack at 0.68 of the crack load of RC control beam.

Hence, CNT beam to RC beam first crack load is: 1.35, 1.09 and 0.84, for room temperature and 400 °C for 1 and 2 hours respectively. Exposure to 600 °C for 2 hours has a dramatic effect on first crack load, reaching 0.45 from CNT- beam first crack load at ambient temperature. This can be attributed to the conversion of the CNT to CO2 at high temperature through the reaction of carbon with oxygen from the atmosphere.

**Figure 10:**
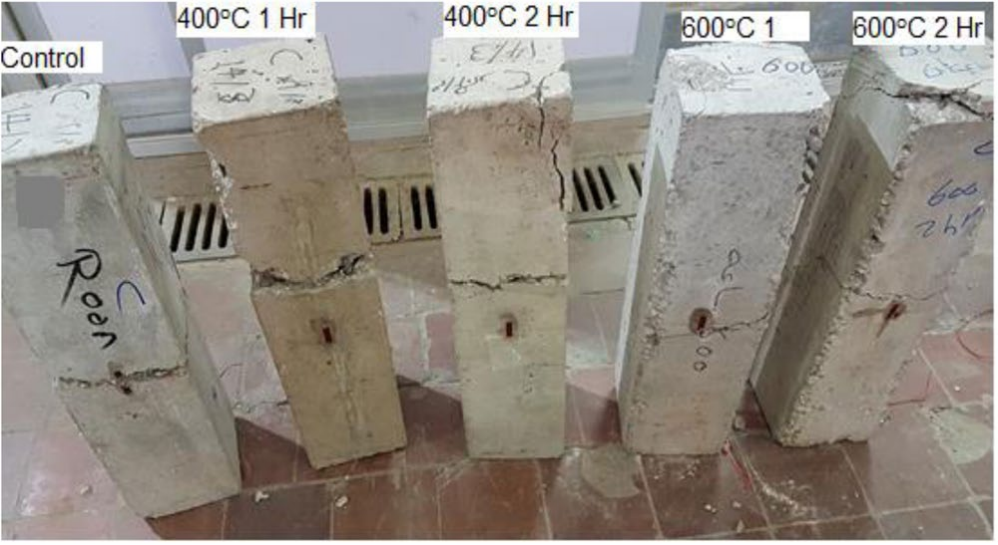
Cracks and failure mode for control beams after fire exposure.

**Figure 11:**
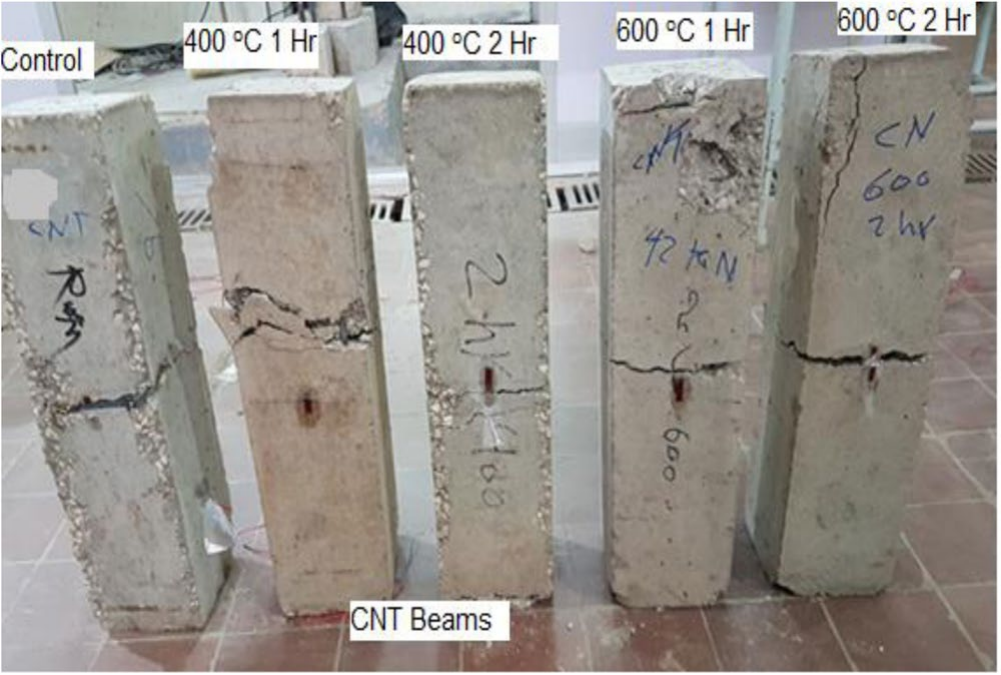
Cracks and failure mode of control beams with CNT after fire exposure.

Figures 10, 11 show the crack pattern of different RC and CNT beams respectively. For all tested specimens the first crack occurred at the beam center, hence, extending to failure. From the photos it can be seen that all beams undergone pure flexure failure, although beams subjected to 600 °C suffered local fractures at the end supports. It is concluded from the photos that CNT limited the crack size at both-normal conditions, and after exposure to 400 °C (one and two hours). On the contrary after reaching 600 °C, voids resulting from burnt CNT led to a more obvious crack.

### Three point load deflection patterns of CNT and RC beams, pre and post elevated temperature exposure

**Figure 12:**
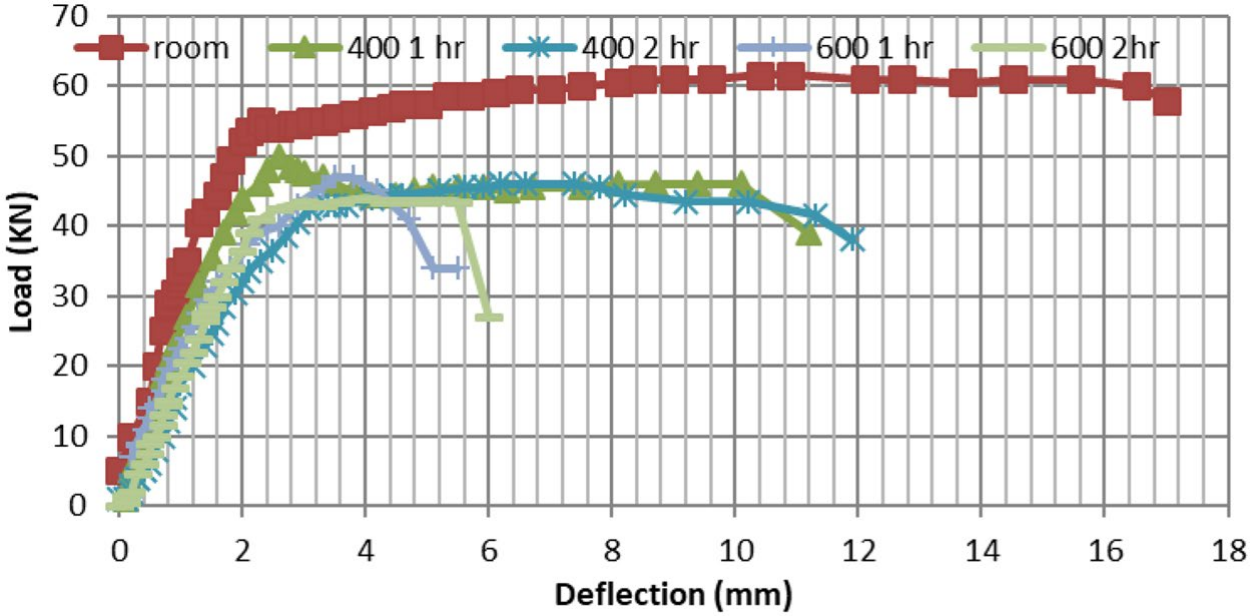
Deflection – load curves post different temperature exposures.

**Figure 13:**
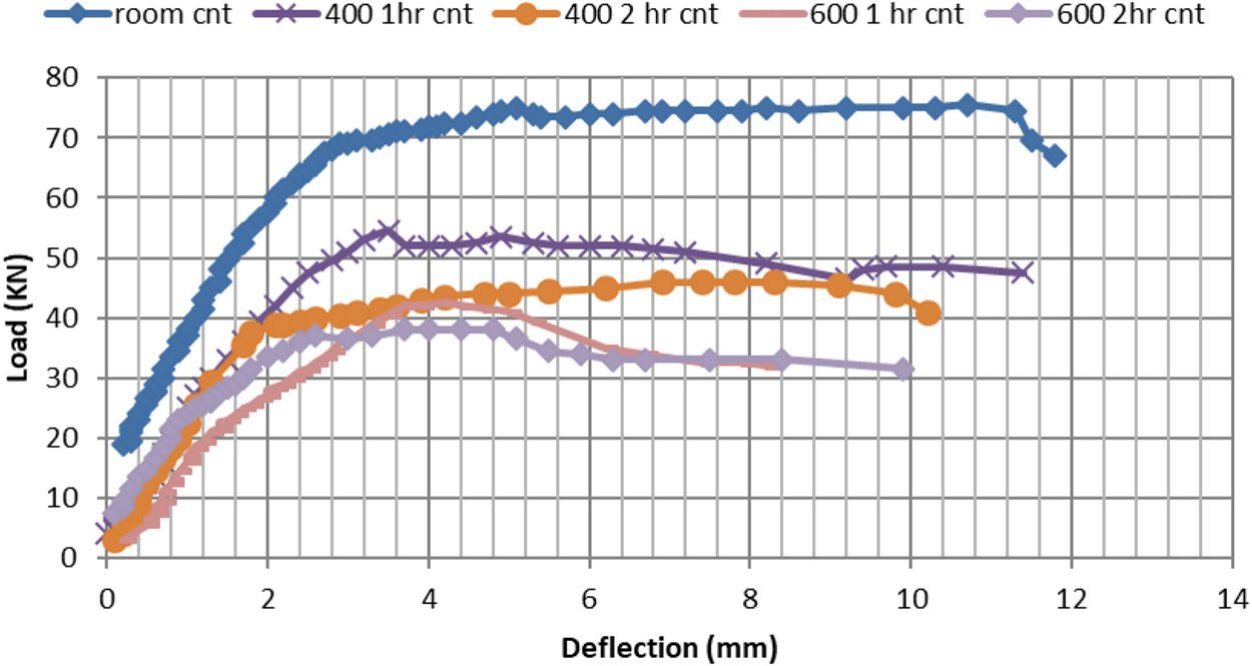
Deflection versus load post different temperatures exposure.

Figures 12, 13 show the load deflection (P-D) curves for all tested beams (RC and CNT respectively). The deflection measured at the beam center. It can be seen from the P-D curve for CNT beams that deformations are almost 71% of deflections of the corresponding RC beams at room temperature, along with higher failure load by about 25%. As for thermally tested beams, exposure to 400 °C for two hours CNT beam maximum deflection is 85%, of the RC beam, while maximum failure load is still higher by 21% compared to RC beam at same temperature conditions. On the other hand, exposure to 600 °C for two hours lead to an extended deflection for CNT beams, exceeding 67% over RC beam deflection, although the maximum load is still higher by 13% for CNT beam.

### Effect of elevated temperature on maximum flexural load of CNT beams

Comparing the reduction in maximum flexural load, for all cases compared to the control reinforced beam For CNT concrete beams (without CNT) at ambient temperature: exposure of CNT beam to 400 °C for one hour, lead to reduction in maximum flexural load by 11%. Extending temperature exposure for 2 hours increased this reduction to 23%. Exposure to 600 °C for one hour, lead to reduction in maximum flexural load 33%. Extending exposure for 2 hours increased this reduction to 39%. At both 400 °C and 600 °C both CNT beams lost half their original strength.

### Shear strains in CNT and RC beams subjected to single point flexural loading, pre and post elevated temperatures exposure

**Figure 14:**
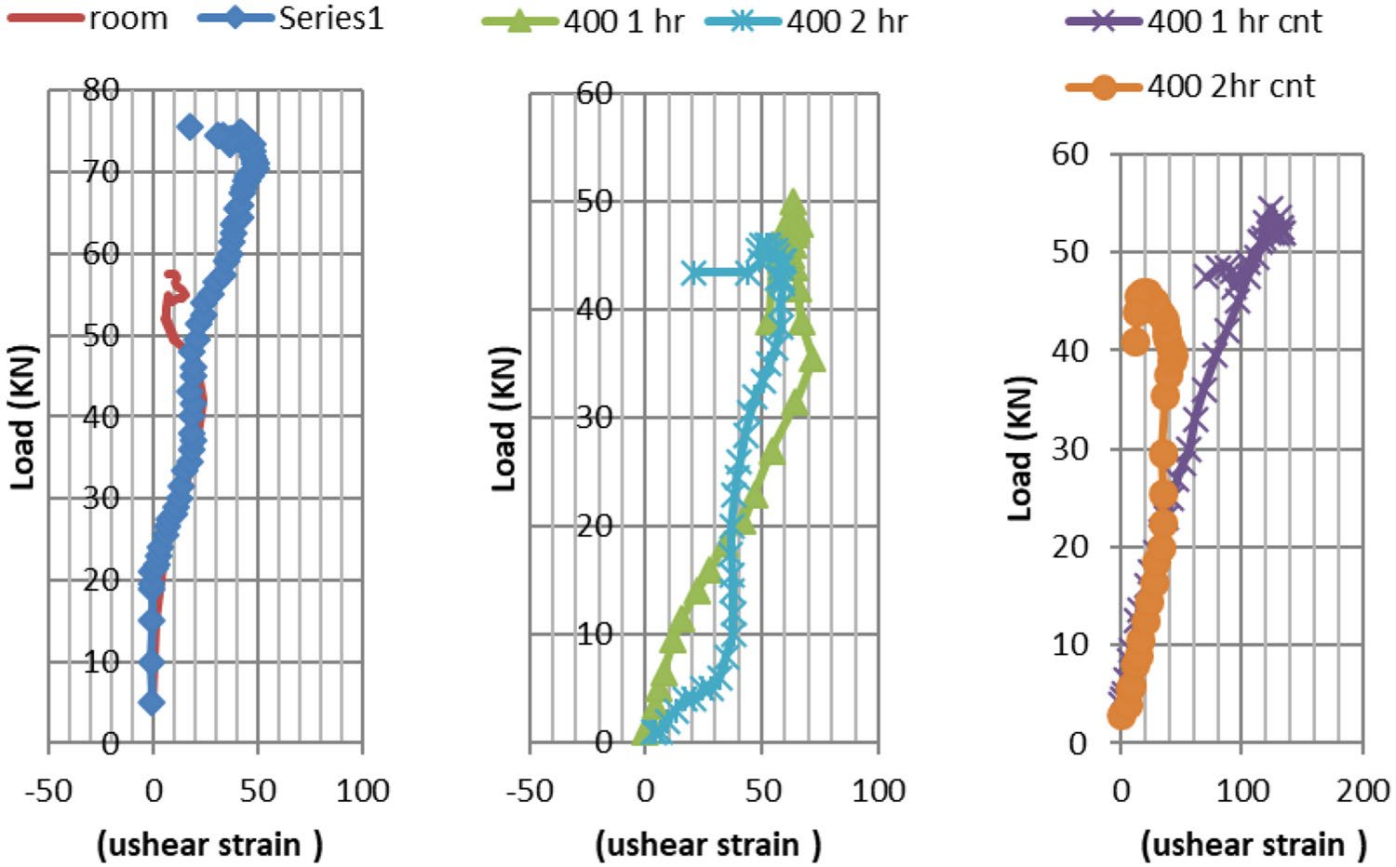
Shear Strains – load curve at ambient temperature versus post exposure to 400 °C, for RC and CNT beams.

As for shear strains CNT beams expressed higher shear strains Fig. 14 compared reinforced concrete at ambient temperature, due to the higher failure load. One hour exposure to 400 °C CNT beams expressed double the shear strain of RC beams. Doubling fire exposure duration at 400 °C lead to doubled shear strains as well. Two hours exposure 400 °C lead to similar strains for both RC and CNT beams.

**Figure 15:**
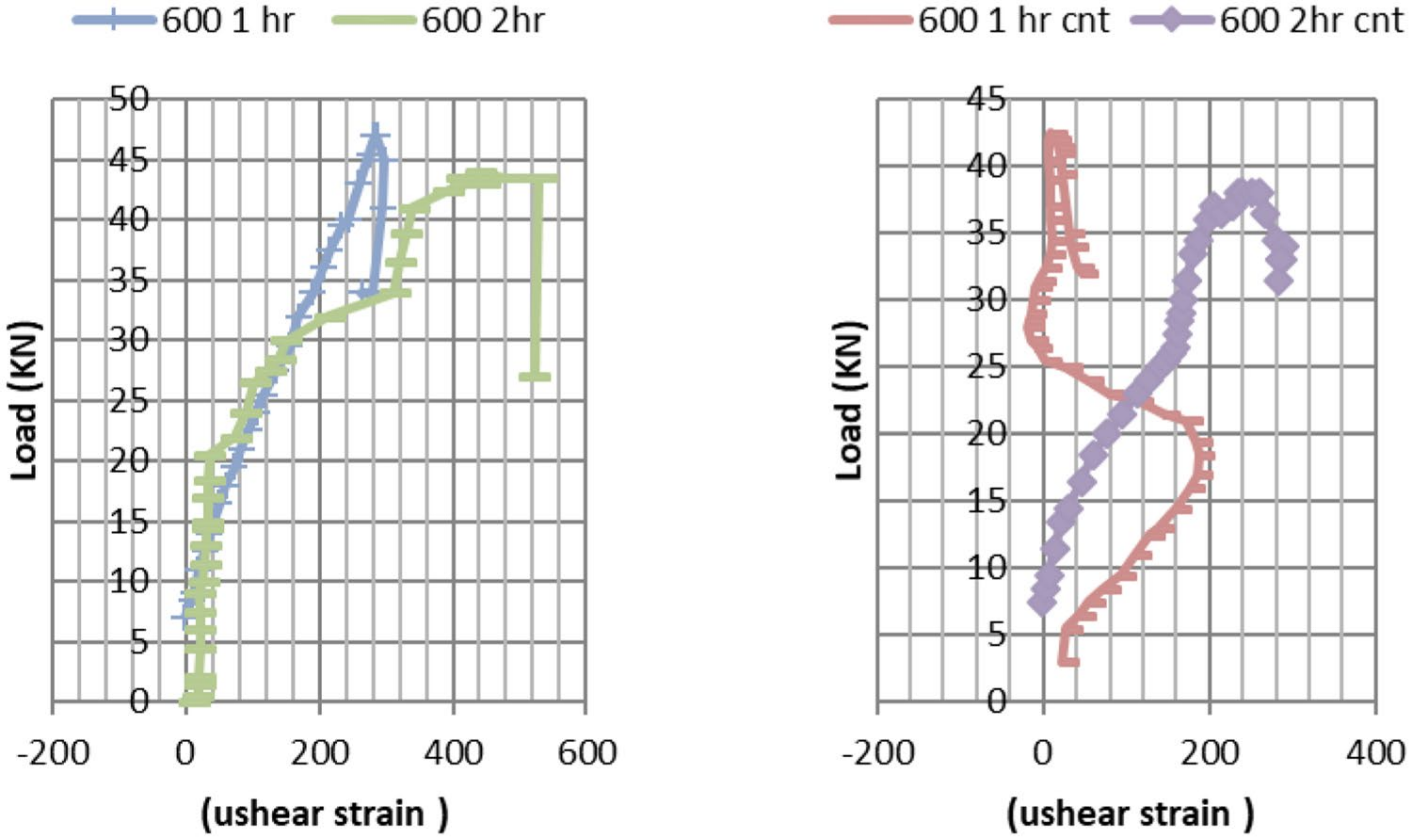
Shear Strains- load curves at ambient temperature versus post exposure to 600 °C, for RC and CNT beams.

It is worth notice that for 600 °C one hour CNT beams Fig. 15, the obvious increase in flexure crack, resulted in relief in the maximum shear strain leading to the shown pattern.

The maximum shear strain for CNT beam is 71% of the RC beam for 600 °C fire tests two hours exposure, CNT shear strains are 67% from RC beams strains.

### Ductility index for CNT and RC beams

**Figure 16:**
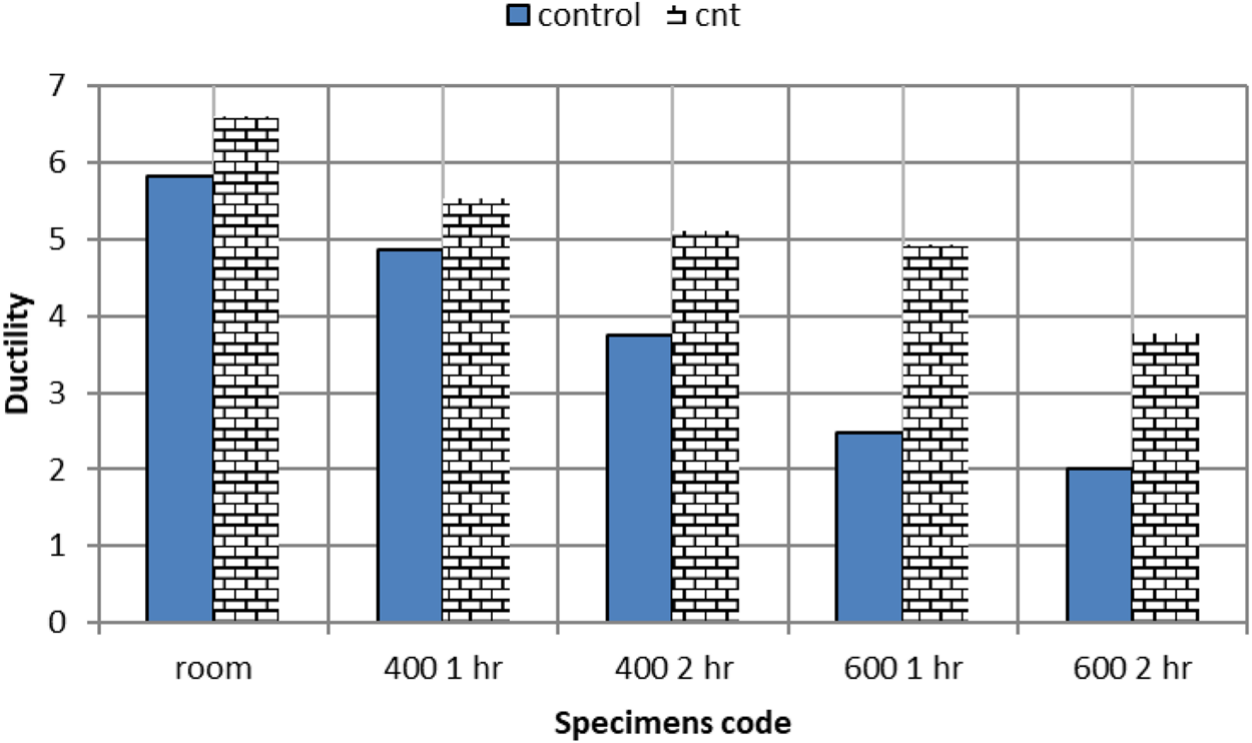
Ductility index of CNT versus RC beams after different temperatures exposure.

Figure 16 displays that the deterioration in ductility curve is steeper for RC beams than CNT ones Control reinforced concrete beams, suffered gradual loss in ductility, almost half the ductility was lost after being exposed to 400 °C for two hours.

On the other hand ductility of CNT reinforced concrete beams improved by about 30% from normal temperature conditions, on exposure to 400 °C for two hours, then ductility was lost after 2 hours exposure to 600 °C.

The mechanical characterization of all tested CNT and RC beams is summarized in Table 3. Superior behavior of CNT beams is clear at normal temperature. CNT beams maintained higher ductility index after all high temperature exposure, for all time durations.

**Table 3:** Mechanical characterization of tested CNT and RC beams.

Specimens	P_u_ (KN)	P_c_ (KN)	D_U_	D_Y_	P_U_/P_c_	D_U_/D_Y_ Ductility index
RC -room	62	44	14	2.4	1.4	5.83
RC-400 1 hr	50	42	11.2	2.3	1.19	4.87
RC-400 2 hr	46	34	12	3.2	1.35	3.75
RC-600 1 hr	47	34	5.2	2.1	1.38	2.48
RC-600 2 hr	44	30	5.6	2.8	1.47	2
CNT-room	76	59	11.9	1.8	1.28	6.61
CNT-400 1 hr	55	48	11.6	2.1	1.15	5.52
CNT-400 2 hr	47	37	10.2	2	1.27	5.1
CNT-600 1 hr	43	32	8.4	1.7	1.35	4.94
CNT-600 2 hr	38	26	9.8	2.6	1.46	3.77

Where Pu failure load, Pc cracking load.

Du ultimate deformation, Dy yield deformation.

## Conclusions

The presented is a pioneer research project investigating the elevated temperatures effect on CNT reinforced concrete beams. Discussed results of testing groups of CNT-RC beams lead to the following conclusions:

### Flexural Strength of CNT Beams

CNT noticeably improved the flexure strength of concrete at normal temperature conditions, through improving both: the load carrying capacity, and ductility index. The Nano-scale characteristics of CNTs shall interact most intimately with CSH. Besides, the considerably huge surface energy and large number of atoms on the nanotube surface shall enhance the interface with C-S-H, generating bridges that enable the CNTs to increase the flexural strength of the cement composites and improve the beams ductility^19,20^.

- Adding a ratio of 0.05% CNT from cement weight lead to an increase of 25% in the load carrying capacity of the tested beams.

- At normal temperature conditions, CNT controlled shear strains, along with the higher load capacities.

### Post Fire Behavior of CNT Beams

Although using CNT in RC beams has a positive impact on their flexural crack resistance, exposure to high temperature for long duration (reaching 2 hours) lead to a negative effect on this crack resistance, consequently applying CNT in concrete needs special attention -if intended to be used in buildings susceptible to fire hazards. This negative effect reaches an extreme at 600 °C, as discussed in details later.

- Using CNT in RC beams increased the first crack load by 35% compared to regular RC beams at ambient temperature. This enhancement was limited to 9% on increasing temperature for 400 °C for one hour exposure on the other hand the beam lost 16% from its first crack load if the duration extended to 2 hours.

- Two hours exposure to 600 °C has a catastrophic effect on the deterioration of the first crack load, reaching less than half the recorded value for the same beam at ambient temperature.

- It can be confidently concluded that, high thermal exposure significantly affected the flexural strength of CNT reinforced beams. Although all beams still maintained higher failure load than the corresponding RC beams except at 600 °C two hours exposure, CNT tolerated less maximum load than the corresponding RC -same conditions by 14%, this can be attributed to the complete combustion of CNT inside the beams, and creation of corresponding voids.
